# El estrés crónico como mediador de la relación entre la posición socioeconómica y el cumplimiento del tratamiento farmacológico de pacientes hipertensos

**DOI:** 10.7705/biomedica.4780

**Published:** 2020-06-30

**Authors:** Deivis Nicolás Guzmán-Tordecilla, Andrés Ignacio Vecino-Ortiz, Diego Lucumí, Graciela Mentz

**Affiliations:** 1 Escuela de Gobierno, Universidad de los Andes, Bogotá, D.C., Colombia Universidad de los Andes Escuela de Gobierno Universidad de los Andes Bogotá, D.C. Colombia; 2 Instituto de Salud Pública, Pontificia Universidad Javeriana, Bogotá, D.C., Colombia Pontificia Universidad Javeriana Instituto de Salud Pública Bogotá, D.C. Colombia; 3 Department of International Health, Johns Hopkins Bloomberg School of Public Health, Baltimore, MD, USA Department of International Health Johns Hopkins Bloomberg School of Public Health, Baltimore, MD USA; 4 Department of Health Behavior and Health Education, University of Michigan, Ann Arbor, MI, USA Department of Health Behavior and Health Education University of Michigan Ann ArborMI USA

**Keywords:** estrés psicológico, hipertensión, cumplimiento del tratamiento, cumplimiento de la medicación, clase social, Colombia, Stress, psychological, hypertension, treatment adherence and compliance, medication adherence, social class, Colombia

## Abstract

**Introducción.:**

La hipertensión arterial sistémica es un problema de salud pública en el mundo. En Colombia, su prevalencia es del 25 % y la mortalidad es alta. Los factores psicosociales que afectan el cumplimiento del tratamiento farmacológico no han sido estudiados suficientemente. En otros países, se ha estudiado el papel del estrés crónico en la relación entre la posición socioeconómica y el cumplimiento del tratamiento farmacológico antihipertensivo.

**Objetivo.:**

Examinar el papel del estrés crónico como mediador de la relación entre la posición socioeconómica y el cumplimiento del tratamiento farmacológico, en pacientes hipertensos de 45 a 70 años en el 2015 y el 2016.

**Materiales y métodos.:**

Se hizo un estudio transversal de una población de pacientes hipertensos. Los datos provienen de la muestra seleccionada para el programa “De todo corazón” en Bogotá, Medellín y Quibdó. El análisis estadístico de los datos se hizo mediante análisis factorial y regresiones multivariadas.

**Resultados.:**

Los resultados confirmaron una asociación positiva entre la posición socioeconómica y el grado de cumplimiento del tratamiento farmacológico, y una relación negativa entre la primera y el estrés crónico. Además, se evidenció que el estrés tiene una asociación negativa con el grado de cumplimiento.

**Conclusiones.:**

Los resultados sugieren que el estrés no es un mediador entre la posición socioeconómica y el cumplimiento del tratamiento farmacológico antihipertensivo en Colombia. Se requieren estudios adicionales para confirmar estas relaciones con una muestra más amplia.

## Abstract (en)

**Introduction:**

High blood pressure is a public health problem worldwide. In Colombia, its prevalence is 25% with a high mortality rate. The psychosocial factors affecting pharmacological adherence among patients have not been sufficiently studied and despite international evidence on their impact, in Colombia, there is a paucity of research on the role of chronic stress in the relationship between socioeconomic status and pharmacological adherence.

**Objective:**

To examine the role of chronic stress in the relationship between socioeconomic status and pharmacological adherence in hypertensive patients aged 45 to 70 years old in three Colombian cities between 2015 and 2016.

**Materials and methods:**

We conducted a cross-sectional study in a population of hypertensive patients. Data for this study came from the first wave of longitudinal study aimed at examining social factors associated with the control of hypertension in Bogotá, Medellín, and Quibdó. Patients with hypertension were selected randomly from a sample of those participating in the hypertension control program *De todo corazón*. For the statistical analysis of the data, we used factorial analysis and multivariate regressions.

**Results:**

We found a positive association between socioeconomic status and the degree of pharmacological adherence and a negative one with chronic stress. Besides, evidence was found that stress has a negative association with the degree of adherence.

**Conclusions:**

The results suggest that stress is not a likely mediator between socioeconomic status and the pharmacological adherence of hypertensive patients in Colombia. Additional studies are required to confirm these relationships with a larger sample.

La hipertensión arterial sistémica se considera un problema de salud pública a nivel mundial ^1^^,^^2^. Se estima que su prevalencia es mayor en Latinoamérica (39,1 %) que en otros partes del mundo (32,3 %) ^3^. Del total de personas hipertensas en el mundo, 9,4 millones mueren cada año, lo que convierte esta afección en el principal factor de riesgo de la enfermedad cardiovascular, con el 31 % (17,5 millones) del total de las muertes reportadas atribuibles a la misma ^4^^,^^5^. Además, se prevé que, en el 2025, habrá 1.500 millones de hipertensos en todo el mundo ^2^.

También en Colombia, la hipertensión arterial sistémica es un problema de salud pública. La prevalencia estimada a partir de una muestra poblacional de adultos fue de 25 % ^6^ y, en el 2015, se informó que había sido la causa básica de muerte de 5.157 personas ^7^. Esta cifra podría ser mayor, ya que se ha establecido que la hipertensión arterial es responsable, aproximadamente, del 45 % de las muertes por cardiopatías y del 51 % de aquellas por accidente cerebrovascular ^4^.

Se ha encontrado que, en general, las personas con hipertensión arterial sistémica poco cumplen con el tratamiento farmacológico ^1^^,^^8^^-^^10^, lo que se traduce en su control deficiente, y en disminución de la calidad de vida y de la supervivencia de quienes la padecen.

Según Gasperin, *et al.*, los pacientes que logran acceder al tratamiento farmacológico tienen un alto riesgo de abandonarlo a largo plazo, debido a múltiples factores psicosociales y a la naturaleza asintomática de la enfermedad ^1^, lo que explicaría el poco control (30 %) de la hipertensión arterial en el mundo ^11^. Se ha establecido, asimismo, que las personas que más incumplen el tratamiento y menos controlan su presión arterial son las que tienen una posición socioeconómica más baja ^11^^,^^12^.

La evidencia acumulada ha demostrado que la posición socioeconómica está asociada con los resultados en salud ^13^^-^^15^. Grotto, *et al.*, explican que esta puede influir en los comportamientos en salud y en el acceso a los servicios de salud o los medicamentos antihipertensivos ^13^. Además, constituye un factor que expone a las personas a condiciones de gran demanda psicológica y de estrés crónico ^13^^-^^18^. En Colombia, una significativa proporción de la población muestra condiciones socioeconómicas como bajo nivel educativo, elevada informalidad laboral y bajos ingresos ^19^^-^^20^, todo lo cual podría contribuir a exponerla al estrés crónico. Diversos autores han encontrado que la hipertensión arterial sistémica está asociada con el estrés ^1^^,^^21^^-^^23^. La exposición al estrés crónico no solo tiene efectos fisiológicos, sino que también genera comportamientos de riesgo para la salud, entre los que se encuentra el incumplimiento del tratamiento antihipertensivo ^24^^,^^25^.

El efecto del estrés crónico en el cumplimiento del tratamiento antihipertensivo farmacológico puede explicarse parcialmente por la carga cognitiva y emocional a la cual se enfrentan las personas con dicha condición. Es posible que al verse abrumadas por múltiples tareas diarias, opten por priorizar actividades más inmediatas, entre las que no se incluye la toma de los medicamentos antihipertensivos ^23^^,^^26^.

Sin embargo, la información publicada sobre el papel del estrés crónico como mediador de la relación entre la posición socioeconómica y el cumplimiento del tratamiento antihipertensivo farmacológico en Latinoamérica, es escasa ^27^^-^^30^. En Colombia, los estudios publicados se han centrado en aclarar la forma en que el estrés predispone a esta condición o a su falta de control ^31^^-^^35^.

En ese orden de ideas, este estudio espera contribuir al conocimiento científico disponible en Colombia y Latinoamérica sobre la relación entre el estrés crónico y el cumplimiento del tratamiento farmacológico antihipertensivo, explorando el papel del primero como mediador entre la posición socioeconómica y el cumplimiento del tratamiento en pacientes hipertensos de 45 a 70 años de tres ciudades de Colombia, en el 2015 y el 2016.

## Materiales y métodos

### Muestra

Los datos de este estudio provienen del programa “De todo corazón” desarrollado por el Grupo de Investigación de Factores Psicosociales en Psicología Clínica y de la Salud de la Universidad de los Andes. Este programa incluyó pacientes de 45 a 70 años de edad vinculados a programas de hipertensión arterial en Bogotá, Medellín y Quibdó. Estas ciudades difieren en términos de niveles de pobreza, oferta de servicios e infraestructura de servicios de salud, especialmente al comparar Quibdó con las otras dos ciudades ^36^^-^^38^.

“De todo corazón” es un programa de carácter longitudinal realizado en dos etapas que utiliza métodos mixtos. En la primera etapa, se seleccionaron las tres ciudades (Bogotá, Medellín y Quibdó) mediante muestreo de criterio o propósito de tipo heterogéneo, con el fin de examinar las variaciones o similitudes del fenómeno en diferentes contextos ^39^. En la segunda etapa, se seleccionaron dos entidades administradoras de planes de beneficio en cada ciudad: una con mayor cobertura en el régimen contributivo y, otra, en el subsidiado. Otros criterios preliminares para la selección de estas entidades fueron el contar con programas de hipertensión arterial, tener una cobertura igual o superior al tamaño de la muestra en cada ciudad, poseer un registro actualizado de las personas que formaban parte del programa y la disposición de participar en el estudio.

Por último, los participantes se seleccionaron por muestreo probabilístico estratificado basado en la distribución de edad y sexo, lo que permitió tener una muestra representativa de individuos dentro de cada entidad administradora de planes de beneficio con el mínimo sesgo de selección.

Los criterios de exclusión para los participantes fueron ser pacientes con diagnóstico concomitante de diabetes mellitus o con alguna discapacidad cognitiva, neurológica, motora o psiquiátrica, que les impidiera la adecuada resolución de las pruebas psicológicas, así como no hablar español.

En el estudio se tomó como muestra la primera ola del programa, conformada por 258 pacientes reclutados entre el 2015 y el 2016. En los análisis que se describen a continuación, se usaron los pesos muestrales desarrollados para la primera ola para incorporar elementos como la falta de respuesta y garantizar que, dentro del rango de edad del estudio, la muestra reflejara las características de edad y sexo de la población colombiana.

El programa *“*De todo corazón” fue aprobado por el Comité de Ética de la Universidad de los Andes mediante el acta 531 del 2015, y el presente estudio fue aprobado y categorizado como estudio sin riesgo por el Comité de Ética de la Escuela de Gobierno Alberto Lleras Camargo de la misma universidad.

#### Instrumentos

La información sobre las variables de interés se obtuvo mediante cuestionarios estandarizados. Para conocer las dimensiones de tales cuestionarios, se hicieron análisis factoriales con los pesos muestrales estimados por el programa ^40^.

#### Cumplimiento del tratamiento farmacológico

Se usó la escala de cumplimiento del tratamiento farmacológico de Morisky ^41^, la cual fue creada para medir el cumplimiento del tratamiento farmacológico en pacientes hipertensos mediante la respuesta a cuatro preguntas dicotómicas sobre la toma de medicamentos por parte de los pacientes y las circunstancias en las que dejan de tomarlos ^42^. Esta escala se ha validado en Brasil, Perú y España, países de habla española o con contextos similares al de Colombia ^43^^,^^44^. En este estudio, el alfa de Cronbach para dicha escala fue 0,54, por lo cual se decidió crear un índice a partir de las preguntas, como se ha hecho en otros estudios ^8^^,^^10^.

Las preguntas de la escala son las siguientes: 1) ¿Alguna vez olvida tomar la medicación para la hipertensión arterial? 2) ¿Algunas veces olvida tomar los medicamentos a las horas indicadas? 3) Cuando se siente mejor, ¿a veces deja de tomar la medicación? y, por último, 4) Cuando se siente mal ¿a veces deja de tomar la medicación? Las opciones de respuesta eran “sí” o “no”, y se asignaba un punto a cada respuesta afirmativa y cero a cada respuesta negativa. Se consideró como pacientes que cumplían el tratamiento a aquellos que respondieron negativamente a todas las preguntas, y que lo incumplían, a aquellos que respondieron a una o más preguntas afirmativamente*.*

#### Estrés crónico

Para medir el estrés crónico se usó la escala de Cohen, *et al.*^45^, con la cual se evalúa el nivel de estrés percibido por las propias personas durante el último mes. Consta de 14 ítems cuyas respuestas se califican en una escala de cinco puntos ^45^. En este estudio, se obtuvo un alfa de Cronbach de 0,85, lo que se considera como indicativo de buena fiabilidad. Además, en el análisis factorial se determinó un único factor con un valor propio (*Eigenvalue*) de 6,4 que acumuló el 45 % de la varianza de la escala. Este mismo cuestionario se validó en Colombia con un alfa de 0,87 y el análisis factorial mostró dos factores (afrontamiento y percepción del estrés) que explicaron el 49,6 % de la varianza ^46^. Además, se ha encontrado que la escala de Cohen tiene una relación significativa con los niveles de cortisol en el cabello ^47^, el cual es un biomarcador que ha demostrado ser confiable para medir el estrés crónico ^48^^-^^50^.

#### Posición socioeconómica

Para las variables de posición socioeconómica, se usó la versión larga del cuestionario “Nivel socioeconómico en la vida adulta” propuesto por la Red MacArthur en el 2009 para medir el estatus socioeconómico y la salud, el cual consta de 12 preguntas ^51^. De él se tomaron las variables de nivel educativo y posición en la comunidad, la primera de estas categorizada como “sin primaria, primaria, bachillerato y más de bachillerato”, en tanto que la variable de posición en la comunidad se usó de forma continua.

Para establecer la variable de posición en la comunidad, se les indicaba a los participantes que seleccionaran en una escalera de diez peldaños aquel en el que consideraban que se encontraban, correspondiendo los más altos a una posición más alta en la comunidad y los más bajos a una más baja.

#### Covariables

La información sobre las covariables también se obtuvo utilizando cuestionarios estandarizados. Para la variable de apoyo social, se utilizó el cuestionario *Medical Outcomes Study-Social Support Survey* (MOS- SSS), escala de Sherbourne, *et al.*^52^ ya validada en Colombia ^53^. Este cuestionario consta de 20 ítems y evalúa cuatro componentes: apoyo emocional e informativo, apoyo instrumental, interacción social positiva y apoyo afectivo, y puede usarse como una escala global que reúne todos estos componentes. En el análisis factorial, se encontró que el primer componente tenía un valor propio de 12,8 y acumulaba el 68 % de la varianza, con un alfa de 0,96. Para efectos de este estudio, se usó la escala de forma global.

Otras covariables del estudio fueron la etnicidad asignada por observación (minorías y no minorías), el sexo (hombres y mujeres), la edad (medida de manera continua en años cumplidos) y la ciudad de residencia del participante (Bogotá, Quibdó o Medellín).

#### Análisis estadístico

Inicialmente, se hizo un análisis descriptivo seguido de uno bivariado mediante regresiones simples entre las variables de posición socioeconómica y estrés crónico, lo que permitió conocer la distribución y la asociación del estrés crónico con las variables de posición socioeconómica. En segundo lugar, se utilizaron funciones *logit* multivariadas para evaluar el efecto mediador del estrés crónico entre la posición socioeconómica y el cumplimiento del tratamiento farmacológico, teniendo en cuenta los criterios de mediación propuestos anteriormente ^54^^,^^55^.

Freedman y Schatzkin propusieron dos pasos para explorar la mediación mediante regresiones ^56^: 1) usar un modelo con el predictor (X) y la variable de resultado (Y): X -> Y, y 2) usar un segundo modelo con la variable predictora, la de mediación (M) y la de resultado: X->M->Y para, posteriormente, comparar el coeficiente de X en ambos modelos, esperando encontrar una disminución de la significación estadística y del efecto entre el predictor y la variable de resultado, esto en caso de presentarse una mediación. Los dos modelos se ajustaron con las covariables descritas previamente.

Para la selección de los modelos, se utilizaron los criterios de información bayesianos y de Akaike; asimismo, se estimó el factor de inflación de la varianza y, por último, se usaron errores estándar consistentes.

Los niveles de significación se determinaron a partir de un valor de p menor o igual a 0,1 dadas las características de medición de las variables de interés y el tamaño de la muestra ^57^. Todos los análisis se hicieron con el programa Stata 14™.

#### Resultados

La muestra estudiada incluyó 258 pacientes hipertensos, 161 mujeres y 97 hombres, de los cuales el 33 % residía en Bogotá, el 37 % en Medellín y el 30 % en Quibdó. El 39 % de los pacientes pertenecía a minorías étnicas.

En cuanto al cumplimiento del tratamiento, el 73 % no lo cumplía; más del 60 % de los participantes no tenía estudios universitarios y el 48 % no estaba empleado. La media de edad fue de 58 años, con un promedio de 16,62 y 78,37 para las escalas de estrés crónico y apoyo social, respectivamente. La variable de posición en la comunidad tuvo una media de 5,097 (cuadro 1).

**Cuadro 1:** Datos descriptivos de los pacientes hipertensos participantes de Bogotá, Medellín y Quibdó

Variables	n	Media o porcentaje (%)
Cumplimiento del tratamiento	250	
No	182	73
Estrés	258	16,62 (DE=8,75; rango: 0 a 42)
Educación	258	
Sin primaria	49	19
Primaria	59	23
Bachillerato	57	22
Más que bachillerato	93	36
Posición en la comunidad	257	5,09 (DE=2,40; rango: 1 a 10)
Sexo	258	
Hombre	98	38
Edad	258	58 (DE=6,46; rango: 5 a 70)
Etnicidad	258	
No perteneciente a minorías	157	61
Minorías	101	39
Ciudad	258	
Bogotá	85	33
Medellín	96	37
Quibdó	77	30
Apoyo social	258	78,37 (DE=17,77; rango: 0 a 95)

Nota: en la columna de “media o porcentaje (%)” se presentan en paréntesis las desviaciones estándar (DE) y los rangos de las variables continuas.

#### Regresiones simples

*Distribución del estrés según las variables de posición socioeconómica.* En cuanto a la distribución y asociación del estrés con la posición socioeconómica (cuadro 2), se encontró que las personas con mayores niveles educativos, es decir, bachillerato y por encima de bachillerato, tuvieron, respectivamente, puntajes promedios de estrés crónico 2,98 y 2,49 mayores que las personas con menor nivel educativo (sin primaria). Por otro lado, por cada peldaño adicional de posición social ocupado en la comunidad, las personas disminuyeron, en promedio, 0,42 puntos en la escala de estrés (p=0,06).

**Cuadro 2:** Distribución del estrés en función de las variables de posición socioeconómica mediante regresiones lineales simples

Variables de posición socioeconómica	Coeficiente β	EE
Educación (categoría de referencia “Sin primaria”)		
Primaria	1,61	1,69
Bachillerato	2,98*	1,7
Más de bachillerato	2,49*	1,55
Posición en la comunidad	-0,42*	0,22

EE: error estándar

*p≤0,1; **p≤0,05; ***p≤0,01

La variable dependiente de los modelos fue la escala de estrés. Estos modelos se utilizaron sin pesos muestrales. La variable de posición en la comunidad se midió en una escalera de uno a diez peldaños, en la que los más altos correspondían a una mejor posición en la comunidad.

Los modelos multivariados finales (cuadro 3) se eligieron teniendo en cuenta los criterios de bondad de ajuste tradicionales: AIC (*Akaike Information Criterion*) y BIC (*Bayesian Information Criterion*), y la prueba de multicolinealidad fue menor de 10.

**Cuadro 3:** Resultado del modelo de ecuaciones estructurales para las variables

**Variables (variable de referencia)**	Model 1(Cumplimiento)		Modelo 2 (Cumplimiento)	
	(OR)	(EE)	OR	(EE)
Estrés			-0,05***	0,01
Educación (Sin primaria)				
Primaria	0,35	0,35	0,48	0,38
Bachillerato	0,11	0,41	0,33	0,44
Más que bachillerato	0,18	0,38	0,40	0,42
Posición en la comunidad	-0,07	0,06	-0,11*	0,06
Sexo (Mujer)				
Hombre	-0,21	0,26	-0,30	0,25
Edad	0,02	0,01	-0,20	0,01
Etnicidad (No perteneciente a minorías)				
Minorías	0,24	0,37	0,29	0,35
Ciudad (Bogotá)				
Medellín	-0,49*	0,30	-0,56*	0,31
Quibdó	-1,02**	0,45	-1,08**	0,45
Apoyo social	0,003	0,007	-0,01	0,007

OR: *odds ratio*; EE: error estándar

*p≤0,1; **p≤0,05; ***p≤0,01

Los modelos 1 y 2 tienen como variable de resultado el cumplimiento. El modelo 1 no incluyó la variable estrés. Los dos modelos se ajustaron por sexo, edad, etnicidad, ciudad y apoyo social.

*Modelo 1: posición socioeconómica y cumplimiento del tratamiento farmacológico.* Las personas con menor nivel de educación reportaron menores probabilidades de cumplir el tratamiento. Por ejemplo, la probabilidad de cumplir el tratamiento de aquellos con educación primaria aumentó 0,35 veces en comparación con las personas sin primaria, en tanto que quienes tenían un nivel superior al bachillerato la aumentaron en 0,18 veces; ambas categorías de educación tuvieron un valor de p>0,1. En contraste, en promedio, por cada peldaño de más en la posición en su comunidad, su probabilidad de cumplir el tratamiento disminuyó 0,76 veces (p=0,2).

*Modelo 2: posición socioeconómica, estrés crónico y cumplimiento del tratamiento farmacológico.* La variable de educación mantuvo la misma tendencia encontrada en el modelo 1, es decir, las personas con mayor nivel educativo tenían más probabilidades de cumplir el tratamiento que aquellas sin primaria, con un valor de p mayor de 0,1. En cuanto a la variable de posición en la comunidad, se encontró que, en promedio, por cada peldaño de más en la comunidad la probabilidad de cumplir el tratamiento disminuyó 0,11 veces (p=0,06), lo que denota un aumento en la significación estadística y el coeficiente. En cuanto al estrés y el cumplimiento, se estableció una asociación significativa y negativa (p=0,001), lo que indica que las personas con mayores niveles de estrés crónico eran las que menos cumplían el tratamiento.

Es preciso indicar que la relación entre la variable de nivel educativo y el cumplimiento del tratamiento no presentó cambios en su significación estadística al incluir la variable de estrés; sin embargo, cuando se revisó la relación entre la posición en la comunidad y el cumplimiento del tratamiento en presencia de la variable estrés, se encontró que la significación estadística y el efecto aumentaron, por lo que no se cumplió el supuesto de mediación planteado por Freedman y Schatzkin ^56^.

Por último, tanto en el modelo 1 como en el 2, las personas que vivían en Quibdó y Medellín tenían menores probabilidades de cumplir con el tratamiento (p≤0,1) en comparación con quienes vivían en Bogotá.

## Discusión

En este estudio se examinó el papel del estrés crónico como mediador de la relación entre la posición socioeconómica y el cumplimiento del tratamiento farmacológico en pacientes hipertensos de tres ciudades de Colombia. Hasta donde se pudo constatar, este es el primero de este tipo que se realiza en Colombia. Los principales hallazgos sugieren que el estrés no es un mediador entre la posición socioeconómica y el cumplimiento del tratamiento. Se establecieron asociaciones significativas entre las diferentes variables de interés y la asociación mencionada aumentó al incluir la variable del estrés. Además, hubo algunos hallazgos novedosos que se discuten a continuación.

Se encontró que los participantes con mayor nivel educativo reportaban mayores niveles de estrés, lo que en algunos casos se contrapone y en otros coincide con algunos estudios ^58^^,^^59^: en una muestra de 283 hombres, Landsbergis, *et al.,* establecieron que las personas más educadas presentaban menores niveles de estrés ^58^, en tanto que, en una muestra de 444 mujeres, Garay, *et al.* reportaron que, a mayor educación, mayores los niveles de estrés ^59^, lo que, según los autores, se debe a que muchas mujeres mayores de 40 años en Latinoamérica aún cumplen una doble función social, pues su mayor nivel educativo aumenta sus posibilidades de incorporarse a la fuerza laboral, y ello se suma a las responsabilidades del hogar que muchas de ellas todavía cumplen ^59^. Esta situación podría aumentar la carga cognitiva derivada de las múltiples actividades que deben realizar.

El argumento expuesto por Garay, *et al.*, podría explicar el por qué en el presente estudio los participantes más educados presentaron mayores niveles de estrés, ya que, de los 258 participantes, 161 eran mujeres con una media de edad de 58 años.

En cuanto a la posición socioeconómica subjetiva o posición en la comunidad y el estrés, se determinó que, por cada peldaño adicional en su posición en la comunidad, las personas reportaban menores niveles de estrés crónico, lo que va en la misma línea de lo presentado por Tang, *et al.,* en un metaanálisis ^60^ en el cual concluyeron que las personas que se percibían en una mejor posición en la escala social reportaban mejores resultados en salud.

Por otro lado, aunque los resultados de los dos modelos empleados difirieron en la significación y la magnitud de los coeficientes, las tendencias fueron las mismas en ambos. El resultado derivado de los dos modelos en cuanto a la relación entre la posición socioeconómica subjetiva y el cumplimiento del tratamiento farmacológico contrasta con lo hallado en otros estudios ^61^, ya que, por cada peldaño en la escalera de posición en la comunidad, las personas tuvieron menores probabilidades de cumplir con el tratamiento, en tanto que, en su estudio, Demakakos, *et al.* hallaron que, a mayor posición socioeconómica subjetiva, mejores resultados cardiovasculares ^61^. Por su parte, Tang, *et al.* refieren que el instrumento de MacArthur usado para medir dicha posición podría presentar un sesgo de respuesta ^60^. Por ejemplo, las personas que se clasifican en lo alto de la escalera podrían verse afectadas por el fenómeno de la deseabilidad social, lo que explicaría que quienes menos cumplen con el tratamiento reporten una posición socioeconómica subjetiva alta.

Ahora bien, la razón de que la variable de la posición en la comunidad esté asociada negativamente con la variable de cumplimiento del tratamiento, sería que, cuanto mejor es la posición en la comunidad, mayor es la demanda por elevarla, lo que implicaría tener menos tiempo para tomar los medicamentos y una mayor carga cognitiva, con el consecuente incumplimiento del tratamiento. Las personas con liderazgo en sus comunidades suelen reportar puntajes altos en la escala de posición socioeconómica subjetiva y mayores niveles de estrés ^52^^,^^62^. Por ello, es factible que dicha condición pueda interferir, en alguna medida, con el cumplimiento del tratamiento.

Además de lo ya mencionando, el hecho de tener un nivel educativo más alto resultó en un mejor cumplimiento del tratamiento, resultado similar al del estudio de Chow, *et al.*^11^.

En el presente estudio, la relación entre el estrés crónico y el cumplimiento del tratamiento farmacológico fue negativa, pues los puntajes más altos en la escala de estrés se relacionaron con una menor probabilidad de cumplimiento. Este hallazgo es consistente con lo encontrado por Marshall, *et al.* en una revisión sistemática de estudios en 19 países, en la cual el estrés afectó negativamente dicho cumplimiento ^23^.

En cuanto al hecho de que a mayores niveles de estrés, menor es la probabilidad de cumplir con el tratamiento, diversos autores han explicado que ello puede deberse a que los pacientes con síntomas de estrés serían más propensos a los efectos negativos de los medicamentos dada su condición de irritabilidad o ansiedad ^23^^,^^63^. Otra posible explicación es que las personas con mayores niveles de estrés crónico sean quienes tienen una mayor carga cognitiva, lo que les dificulta priorizar actividades como la toma de los medicamentos en las horas y las cantidades indicadas.

Un hallazgo coincidente en el presente estudio es que el cumplimiento difirió según el lugar de residencia, pues en Quibdó, la ciudad con el menor desarrollo socioeconómico, la probabilidad de cumplir con el tratamiento fue 1,02 veces menor que en Bogotá. Aunque no se encontraron otros estudios que compararan diversas zonas urbanas ^64^, en el país, sí se han reportado diferencias entre lo urbano y lo rural. En su estudio, Camacho, *et al.*, encontraron que las personas residentes en las zonas rurales de Colombia con menor desarrollo socioeconómico cumplían menos con el tratamiento que quienes vivían en zonas urbanas ^65^.

Algunas limitaciones del presente estudio merecen consideración. Primero, la muestra fue limitada en términos de diversidad racial (una sola observación en población indígena); sin embargo, incluso unificando las observaciones en población indígena y afrocolombiana bajo el criterio de minorías étnicas, no se evidenció una asociación significativa. Segundo, el tamaño de la muestra (258 participantes) limitó el poder estadístico para detectar asociaciones significativas, pero de todas maneras se encontraron relaciones significativas entre las variables de interés. Tercero, la información provenía de los propios participantes, incluida la medición de la variable de estrés en una escala cuyas preguntas tenían como referencia el último mes, lo que podría implicar un sesgo de memoria, aunque debe mencionarse que, según diferentes estudios, esta escala tiene similitud con biomarcadores del estrés crónico ^47^^-^^50^.

La valoración indirecta del cumplimiento del tratamiento está relacionada con posibles sesgos de información, como la deseabilidad social. Sin embargo, el cuestionario empleado se creó para valorar el cumplimiento del tratamiento en pacientes con hipertensión arterial y, además, es breve y muy fácil de aplicar, tiene una gran especificidad y un gran valor predictivo positivo, así como poca exigencia para su comprensión, y fue validado en su versión española por Val, *et al.*^43^, y se ha usado previamente en Colombia ^10^.

Por último, debe mencionarse el sesgo de autoselección. Los pacientes que hicieron parte de esta muestra podrían considerarse como los que más cumplían el tratamiento, dadas su disposición para ser parte de un programa de hipertensión arterial y su voluntad de participar en este estudio. En otras personas, el cumplimiento podría ser menor debido a diversos factores psicosociales y biológicos que interferirían en la toma de sus medicamentos.

A pesar de todas las limitaciones, el estudio sugiere que el estrés crónico no es un mediador entre la posición socioeconómica y el cumplimiento del tratamiento farmacológico en pacientes hipertensos inscritos en programas de hipertensión en Quibdó, Bogotá y Medellín, aunque permite plantear la hipótesis de que puede ser un elemento modificador de dicha relación. Estos hallazgos no son concluyentes, por lo que deben considerarse con cautela, ya que la muestra no fue lo suficientemente amplia para el país y las asociaciones significativas se establecieron a partir de un valor de p de 0,1 o menos.

En futuros estudios en el país, deberá contarse con muestras más amplias y representativas y con mayor participación de minorías étnicas, y medir el cumplimiento y el estrés crónico con otros cuestionarios para indagar sobre el mecanismo moderador del estrés en esta relación. Se sugiere, asimismo, incorporar otros grupos poblacionales pertenecientes a diferentes niveles socioeconómicos que no necesariamente estén en programas de hipertensión arterial.

Por otro lado, los proveedores de servicios de salud deberían evaluar sistemáticamente signos de estrés crónico en sus pacientes como un factor de riesgo para el incumplimiento del tratamiento farmacológico.
